# Neurophysiologic Innovations in ALS: Enhancing Diagnosis, Monitoring, and Treatment Evaluation

**DOI:** 10.3390/brainsci14121251

**Published:** 2024-12-13

**Authors:** Ryan Donaghy, Erik P. Pioro

**Affiliations:** 1 Department of Neurology, Feinberg School of Medicine, Northwestern University, Chicago, IL 60611, USA; ryan.donaghy@nm.org; 2 Djavad Mowafaghian Centre for Brain Health, Division of Neurology, Department of Medicine, University of British Columbia, Vancouver, BC V6T 1Z3, Canada

**Keywords:** amyotrophic lateral sclerosis (ALS), motor unit number estimation (MUNE), transcranial magnetic stimulation, biomarker, clinical trial

## Abstract

Amyotrophic lateral sclerosis (ALS) is a progressive disease of both upper motor neurons (UMNs) and lower motor neurons (LMNs) leading invariably to decline in motor function. The clinical exam is foundational to the diagnosis of the disease, and ordinal severity scales are used to track its progression. However, the lack of objective biomarkers of disease classification and progression delay clinical trial enrollment, muddle inclusion criteria, and limit accurate assessment of drug efficacy. Ultimately, biomarker evidence of therapeutic target engagement will support, and perhaps supplant, more traditional clinical trial outcome measures. Electrophysiology tools including nerve conduction study and electromyography (EMG) have already been established as diagnostic biomarkers of LMN degeneration in ALS. Additional understanding of the motor manifestations of disease is provided by motor unit number estimation, electrical impedance myography, and single-fiber EMG techniques. Dysfunction of UMN and non-motor brain areas is being increasingly assessed with transcranial magnetic stimulation, high-density electroencephalography, and magnetoencephalography; less common autonomic and sensory nervous system dysfunction in ALS can also be characterized. Although most of these techniques are used to explore the underlying disease mechanisms of ALS in research settings, they have the potential on a broader scale to noninvasively identify disease subtypes, predict progression rates, and assess physiologic engagement of experimental therapies.

## 1. Introduction

Amyotrophic lateral sclerosis (ALS) is the most common adult motor neuron disease (MND), which is characterized by the relentless loss in varying proportions of both upper motor neurons (UMNs) in the brain and lower motor neurons (LMNs) in the brainstem and spinal cord. The neuropathology of ALS reveals widespread abnormalities of motor neurons and their axons, including degeneration of corticomotoneurons (Betz cells) in the primary motor cortex (layer V) and the LMN pool within the anterior horn of the spinal cord surrounded by reactive astrogliosis [1]. The crude worldwide incidence and prevalence of ALS is estimated at 1.59 per 100,000 person-years and 4.42 per 100,000 population [2]; significant regional variations have been reported [3,4,5,6].

Understanding disease presentation and characteristics has evolved from purely a clinical diagnosis to one supported by electrodiagnostic criteria, although still heavily reliant on the neurologic examination. Current ALS diagnostic criteria include the revised El Escorial Criteria [7,8] supplemented by the Awaji electrodiagnostic modifications [9] as well as the Gold Coast Criteria [10]. These criteria have been well described in the literature and rely upon demonstrating progressive lower motor neuron dysfunction with varying degrees of diagnostic certainty (Table 1). However, these diagnostic criteria are complex to apply, and patients often experience significant delays in diagnosis [11], which may bias MND clinical trials with patients presenting in later stages of disease.

Paralleling the development of categorical diagnostic criteria, functional markers of disease progression have been devised, with attention to patient-reported or physician-assessed symptom burden via the revised ALS-Functional Rating Scale (ALSFRS-R) [12], muscle strength by MRC sum score or hand-held dynamometry, and respiratory function by vital capacity measures [13]. Developing neurophysiologic biomarkers revealing early disease progression or engagement of pathophysiologic targets would assist patient stratification in clinical trials and ultimately generate new trial outcome measures, fulfilling critical needs in the MND field.

From its conception in the 1970s, the goal of using electrodiagnostic techniques to explore the integrity of functional motor units has remained an area of focus in the MND field. Because of degeneration of LMNs and their subsequent incomplete reinnervation, ALS and LMN-predominant MNDs (e.g., progressive muscular atrophy, Kennedy disease) are ideal conditions to use motor-unit number estimation (MUNE) and other electrodiagnostic techniques to describe the complex progressive LMN pathology. Furthermore, neurophysiologic assessment of UMN dysfunction is important for objective characterization of ALS and UMN-predominant MNDs (e.g., primary lateral sclerosis) with transcranial magnetic stimulation (TMS) and other techniques, since current diagnostic paradigms rely almost solely on bedside examination. These and other related electrodiagnostic techniques used to objectively assess abnormalities in ALS of the LMN (from anterior horn cell in brainstem or spinal cord to muscle) and the UMN (from corticomotoneuron to axon terminal contacting anterior horn cell) are the focus of this review. We will also discuss the role of electrodiagnostic assessment of less-often affected peripheral components, including the autonomic and sensory nervous systems.

This review summarizes pivotal historic studies, as well as recent peer-reviewed studies across a variety of novel neurophysiologic parameters applied to clinical MND research. Relevant studies were reviewed from April 2024 to December 2024, with articles represented from 1971 to 2024. The National Institutes of Health Clinical Trials website (clinicaltrials.gov, accessed on 17 November 2024) was also reviewed until November 2024 to describe active and recently closed clinical trials utilizing the neurophysiologic methods detailed in the text.

## 2. Lower Motor Neuron Assessment

### 2.1. Motor-Unit Number Estimation

In neuromuscular medicine, the assessment of a single motor unit and its recruitment pattern requires significant training, mastery of technique, appropriate participation by the patient, and a sense of the rater’s own self-reliability. In the 1970s, electrodiagnostic research groups sought to achieve more objective assessments of the motor unit. McComas et al. derived the first motor-unit number estimation (MUNE) technique, where increasing sub-threshold stimulation of a nerve–muscle pair was used to generate a stimulus–response curve. The averaged incremental increase in compound muscle action potential (CMAP) amplitude response across increasing stimulation intensity approximates the single-motor unit action-potential (SMUP) amplitude; SMUP is defined as an averaged sum of muscle fiber action potential amplitudes, each making up the various single motor units activated over the range of electrical current applied. That SMUP amplitude was divided into the supra-threshold generated CMAP of the same nerve–muscle pair, with the resultant value approximating the number of motor units [14].

The advent of a novel MUNE technique was not without criticism, and later groups challenged the assumptions of the McComas method, including that the earliest recruited SMUP’s have distinct firing levels and whether they represent the population of SMUP’s recruited across the spectrum of increasing muscle contraction [15]. Given the concern that there could be significant alteration of SMUP firing in the earliest recruited motor units, a multi-point stimulation motor unit estimation method was devised to better characterize the population of early motor unit recruitment [15,16]. Multi-point stimulation remains a tool to which more recently developed MUNE techniques are compared (MUNIX, MScanFit as described later in the text), and demonstrates strong reproducibility and ability to differentiate MND patients from health controls [17].

### 2.2. Motor Unit Number Index

The motor unit number index (MUNIX) technique describes an idealized motor unit number estimation from the parameters of the maximal compound muscle action potential (CMAP) of a given muscle and a surface-EMG interference pattern (SIP) across multiple levels of isometric force provided by the patient, yielding an ideal case motor unit count (ICMUC) [18]. The ICMUC is defined by [CMAP Power × SIP Area]/[CMAP Area × SIP Power], which approximates the number of motor units for a given muscle. A further term, the motor unit size index (MUSIX) is derived from the CMAP amplitude divided by the MUNIX. In healthy controls across multiple assessed muscles, ranges of normative values have been tabulated, and MUNIX has correlated well with CMAP amplitude [19,20]. The clinical application of MUNIX has proven to be reproducible in test–retest models with strong inter- and intra-rater reliability [20].

### 2.3. MUNIX as a Diagnostic/Categorical Biomarker

**Figure 1:**
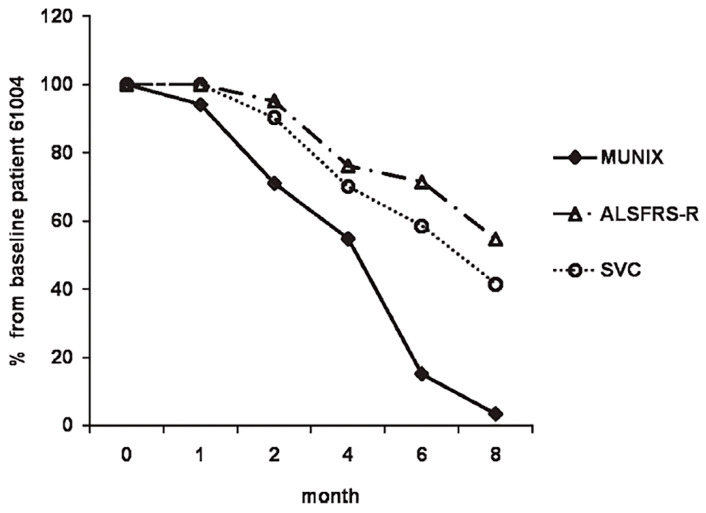
Relative percent drop of the motor unit number index (MUNIX) in a patient with ALS is greater and detected earlier than changes in the revised ALS functional rating scale (ALSFRS-R) score and slow vital capacity (SVC). (Modified and used with permission from reference [19]).

MUNIX values of ALS patients are significantly lower than those of healthy controls, with motor unit loss by MUNIX detected in ALS patients well before a decline in CMAP amplitude can be identified by traditional nerve conduction study (NCS) [21]. MUNIX loss has reliably demonstrated a steeper decline on sequential examinations than currently used physiologic markers of MND severity and progression, including the revised ALS-functional rating scale (ALSFRS-R) and slow vital capacity (SVC) [12,21] (Figure 1), prompting investigators to consider whether MUNIX-based motor unit estimation could serve as an electrodiagnostic biomarker in ALS at multiple phases of clinical trial design. More recently, MUNIX mean scores have been shown to significantly predict more rapid rates of ALS disease progression measured by serial ALSFRS-R responses [22].

Other groups have attempted to enrich the sensitivity and specificity of MUNIX-based testing by incorporating known clinical phenomena seen in ALS, namely the presence of the split-hand pattern, in which CMAP amplitudes are lower in the abductor pollicis brevis (APB) and/or first dorsal interosseous (FDI) muscles than in the relatively spared abductor digiti minimi (ADM) muscle [23,24]. The split-hand MUNIX, [(APB MUNIX × FDI MUNIX)/ADM MUNIX], has been shown to better differentiate ALS patients from healthy controls than a previously described CMAP-based split-hand index and better correlate with the ALSFRS-R [25], as well as to distinguish ALS from MND mimics with a cohort of myopathy, post-polio syndrome, CIDP, and cramp fasciculation syndrome, among other conditions [26]. MUNIX decline has, therefore, been considered a potential biomarker of disease severity at inclusion to a clinical trial, an early sign of disease progression once enrolled in a trial, and ultimately, a stabilization in the decline of MUNIX as a hopeful sign of pharmacodynamic target engagement as a trial outcome [27].

### 2.4. MUNIX as a Marker of Disease Progression/Pharmacodynamic Biomarker

Beyond its use as an adjunct in ALS diagnosis and disease-severity stratification, MUNIX has also been utilized in the assessment of disease progression. Decline in MUNIX of single muscles, as well as the sum of multiple MUNIX values, has correlated well with previously established markers of ALS disease progression, namely the ALSFRS-R [28,29] In some studies, the relative rate of progressive decline in MUNIX values was greater than that of the ALSFRS-R and could even precede the development of clinical objective weakness evaluated by manual muscle testing [30]. Given the apparent early clinical changes in ALS by MUNIX compared with more traditional assessments of motor function, there has been interest in employing this technique as a pharmacodynamic biomarker in ALS clinical trials. Studies have estimated that employment of MUNIX as a defined clinical outcome in trials could reduce both the time needed to assess therapeutic efficacy and reduce the number of participants required to generate statistical significance. The RESCUE ALS investigators used the mean percent change in summed MUNIX score as the primary outcome in their CNM-Au8 gold nanoparticle antioxidant trial in ALS, which, while not demonstrating significant change in MUNIX values between the trial arms, did show that this technique could prove useful in future clinical studies [31,32]. There are many active and recently closed clinical trials employing MUNIX as a secondary outcome with the goal that reduction of MUNIX decline could prove a useful electrodiagnostic biomarker of target engagement [33,34,35].

Use of MUNIX in the clinical assessment of ALS patients has not been without considering its limitations. One of the most common critiques of the MUNIX technique is that it requires the participation of the patient to provide varying levels of force to generate different surface interference patterns on surface EMG, thus making the technique difficult to apply to patients with comorbid cognitive impairment and in non-functional muscle, as seen in later stages of disease [36]. There is also some concern from other groups that MUNIX may not obtain a fully representative sample of motor units [37]. Difficulties also arise with MUNIX when used across multiple clinical trial sites, as survey data suggest that certified MUNIX testers had to undergo on average, three testing sessions to achieve a <20% coefficient of variation prior to participating in the trial, a possible burden to subject enrolment and trial participation [38]. Nonetheless, given the short acquisition time and robust data supporting its use, MUNIX remains a widely applied technique in the MND clinical trial landscape.

### 2.5. MScanFit

Given the questions raised above about MUNIX and its limitations, newer models of motor unit investigation have been designed, with the MScanFit MUNE technique emerging as another prospective electrodiagnostic ALS biomarker. Unlike MUNIX, MScanFit fits a mathematical model to a graded CMAP stimulus–response curve, which presumes to consider the variability of all the motor units recorded over a given muscle and generate a motor unit number estimation [37]. Jacobsen et al. demonstrated that MScanFit reliably differentiated ALS patients from healthy controls better than MUNIX, at the cost of a longer examination time [37]. In serial examinations of both healthy controls and ALS patients, MScanFit had significantly lower coefficients of variation compared to MUNIX and Multi-Point Stimulation techniques [37]. Furthermore, MScanFit demonstrated larger rates of decline per month in ALS patients compared with alternate MUNE techniques, as well as with traditional markers of ALS disease progression including worsening ALSFRS-R scores and CMAP amplitude decline [39]. Adding the characterization of the split-hand index to MScanFit exhibited greater diagnostic sensitivity and specificity than competing split-hand MUNE techniques [26].

Many studies using MScanFit have focused on differentiating ALS patients from healthy controls, which does not reflect the more common clinical scenario of distinguishing ALS from MND-mimicking conditions. However, MScanFit of APB and FDI muscles, as well as the split-hand MScanFit-MUNE technique have reliably differentiated ALS from MND-mimics within a cohort of myopathy, CIDP, post-polio, and cramp-fasciculation syndrome besides other conditions [26]. MScanFit has also been used to shed light on the putative mechanisms of ALS disease pathophysiology, demonstrating that the pattern of neurodegeneration in the distal muscles precedes that of proximal muscles, suggesting a dying-back phenomenon occurring at the level of the lower motor neuron [36]. The clinical trial landscape has begun to employ MScanFit into trial design, with the recently closed trial RESCUE ALS and the active trial RANTAL (NCT06219759) using the technique.

### 2.6. The Neurophysiologic Index and F-Wave Investigations

In parallel to the development of MUNE techniques, other groups have investigated more traditional electrodiagnostic parameters, with efforts focused on incorporating F-wave characteristics into MND biomarkers. The neurophysiologic index (NPI) was developed based on observations that declining CMAP amplitudes, prolonged distal motor latencies, and decreasing frequency of F-wave responses correlated with loss of muscle bulk and clinical weakness. This observation was ultimately described by the formula NPI = [CMAP amplitude/Distal Motor Latency] × (F-wave frequency > 20 recordings) when recording over the ADM muscle [40,41]. The NPI has detected strong significant differences between ALS patients at different King’s stages of disease [41]. This index has also proven to be well correlated with muscle strength and is a more sensitive indicator of clinical decline than more traditional MND biomarkers like FVC and delta-ALSFRS-R [42,43].

Other groups have focused on characterizing F-waves in ALS. Notably, F-wave amplitudes in multiple nerve–muscle pairs are higher in ALS patients compared to healthy controls (so-called “giant F-waves”), and a greater number of identical “repeater F-waves” is noted in the ALS population [44,45]. Submaximally generated F-wave recordings can also be used to estimate responses from a population of single-motor units approximating the SMUP and ultimately generate an F-wave MUNE [46]. This F-wave MUNE technique has been automated and used to differentiate ALS patients not only from healthy controls but also from possible ALS mimics, including remote poliomyelitis [47].

### 2.7. LMN Hyperexcitability

Another characteristic of MND pathophysiology is the concomitant LMN perikaryal/axonal hyperexcitability that generates fasciculation potentials in muscle, as detected by needle electrode examination on routine electromyography to assist in diagnosing ALS. Many properties of axonal hyperexcitability have been explored, most notably the strength–duration time constant (SDTC) reflecting Na^+^ current permissibility and threshold electrotonus (TE) representing axonal membrane accommodation due to K^+^ currents [48]. First described by Bostock and colleagues in 1995, the principle of threshold electrotonus is to evoke a submaximal CMAP at different initial stimulus intensities and add a second polarizing current, which provides a threshold response–time curve reflecting accommodation of the nerve to the polarized current [49]. In this study, ALS patients showed two populations of TE responses in contrast to both healthy and other neurologic-condition controls—a greater threshold reduction than control suggesting hyperexcitability (deemed type I responders) or a rapid increase in threshold representing significant hyperpolarization (type II responders); both pathophysiologies are thought to reflect abnormal K^+^ channel conductance [48,49]. The SDTC describes the rate of stimulus current decline as duration of a threshold stimulation increases, which is dependent partially on Na^+^ channel conductance [48]. Interestingly, the SDTC is increased in ALS patients compared with healthy controls, distinguishing those two groups even in early disease states when CMAP’s are in the normal range [50]. Thus, the identification of electrophysiologic markers of axonal hyperexcitability has both garnered interest in its use as a clinical trial biomarker and as a therapeutic target in MND, given the hypothesized abnormal axonal ion channel conductance described.

### 2.8. Electrical Impedance Myography

While traditional NCS-based and MUNE tools have generated much interest in ALS research, a parallel investigation into the electrophysiologic properties of affected muscle has also garnered attention as a possible biomarker of MND. Electrical impedance myography (EIM) describes the electrophysiologic properties of a given muscle, namely the electrical resistance to low-intensity but high-frequency electrical current, and the phase which is a measure of cell membrane integrity and its ability to alter the application of an alternating current [51]. This tool is non-invasive, quick to use, and relies on the inherent architecture of the underlying muscle. The phase parameter showed promising sensitivity (90%) and specificity (100%) in differentiating ALS patients from healthy controls [52]. Furthermore, the coefficient of variation of EIM when used to track the rate of decline in ALS out-performed that of hand-held dynamometry and the ALSFRS-R [53]. However, EIM parameters are known to be abnormal in other diseases causing neurogenic injury, including motor radiculopathy and carpal tunnel syndrome; longitudinal EIM assessment of ALS patients versus these ALS-mimic conditions did not reveal significant differences between them [54,55,56].

It has been proposed that EIM, when used as a biomarker of therapeutic target engagement, could significantly reduce the number of patients required in future clinical trials when compared with the traditionally used ALSFRS-R [53,57]. Another highlight of the EIM technique is its ability to assess bulbar disease progression, which is more difficult to characterize by traditional clinical measures (i.e., bulbar sub-score of the ALSFRS-R, vital capacity measurements). EIM of the tongues of ALS patients demonstrates reduced phase and increased resistance compared with those of healthy controls [58]. With the advent of improved computational processing, a new technique called Tensor-EIM characterizes a multi-directional assessment of tongue muscle electrical impedance [59]. This tool was projected in a hypothetical clinical trial scenario to reduce the number of needed participants significantly compared with the ALSFRS-R bulbar sub-score in an ALS patient cohort [59].

### 2.9. Single Fiber EMG

While single fiber EMG (SFEMG) is frequently used to investigate diseases affecting the neuromuscular junction (NMJ) (e.g., myasthenia gravis), it also has applications in MND and other diseases resulting from neurogenic injury. With chronic neurogenic injury, reinnervating motor units demonstrate increased jitter and later increasing fiber density reflecting the fiber-type grouping seen on muscle biopsy in neurogenic diseases [60]. As collaterally reinnervated NMJs mature, jitter decreases while fiber density remains increased, suggesting that nascent NMJ transmission is impaired and matures with time [60]. ALS patients are readily differentiated from healthy controls by increased jitter and higher fiber density measurements on SFEMG [61]. In a more common clinical scenario, such SFEMG parameters can distinguish ALS patients and ALS patients with comorbid cervical stenosis from patients with symptomatic cervical stenosis alone [62]. Limitations of SFEMG in ALS include the high level of operator skill required for robust studies, the need for voluntary muscle activation unless performing stimulation-activation SFEMG, and the uncertain specificity in distinguishing ALS from many of its disease mimics [60].

## 3. Upper Motor Neuron Assessment

### 3.1. Transcranial Magnetic Stimulation (Single-Pulse and Paired-Pulse Models)

**Figure 2:**
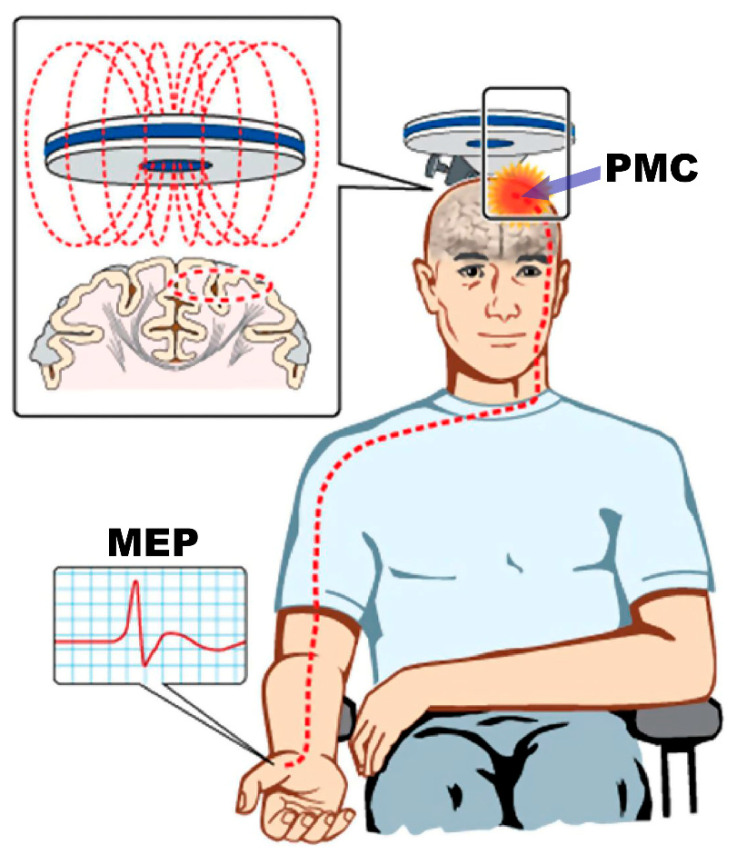
Transcranial magnetic stimulation excites a network of neurons in the underlying primary motor cortex (PMC) with a motor evoked potential (MEP) recorded over a contralateral intrinsic hand muscle (abductor pollicis brevis). (Modified and used with permission from reference [53]).

Though understanding of LMN dysfunction in ALS has been well characterized by traditional and research electrodiagnostic methods, the characteristics of UMN pathophysiology remain challenging. At the time of this report, there is still no electrophysiologic tool to detect UMN dysfunction in routine clinical practice. Transcranial magnetic stimulation (TMS) is a non-invasive method for understanding the pathophysiology of UMN dysfunction by using a targeted magnetic pulse to induce a stimulating electric current within the primary motor cortex. This in turn activates the corticomotoneuron pool and generates a motor evoked potential (MEP) in the relevant contralateral muscle [63,64] (Figure 2) [65].

Within the TMS literature, both single-pulse and multi-pulse paradigms have been used with success in differentiating ALS disease characteristics from controls. Single pulse TMS methods have demonstrated that patients with ALS have MEPs with increased amplitudes and steeper response curves than do healthy controls [66] or disease mimics [67]. Additional studies highlight that the cortical silent period, which is the duration of electromyographic silence following a TMS stimulus, is reduced significantly in ALS compared with both healthy controls and ALS disease mimics [68]. Further work in paired-pulse TMS models by Vucic and colleagues, in which subthreshold conditioning stimuli are delivered at different interstimulus time intervals and the effect of that preconditioning stimulus on the amplitude of a second applied stimulus needed to generate a fixed MEP amplitude (called threshold-tracking TMS) is assessed, has further explored the underpinnings of UMN dysfunction in MND [69].

Two promising threshold-tracking paired-pulse TMS parameters differentiating ALS from controls are short-interval cortical-inhibition (SICI) and intracortical facilitation (ICF). SICI, which is described by the increase in the conditioned stimulus intensity needed to maintain a targeted MEP, is significantly reduced in ALS patients from control subjects, most prominently in patients with spinal-onset ALS [69]. This reduced SICI period in ALS patients is followed by a period of increased ICF, which is manifested by a decrease in the stimulus intensity required to maintain the target MEP amplitude. These findings suggest underlying corticomotoneuronal signaling hyperexcitability [69]. This has been validated by multiple groups in recent years, and that increased cortical hyperexcitability is correlated with shorter survival [70,71] as well as with ALS-associated cognitive impairment [72]. Furthermore, Otani and colleagues combined the measures of elevated SDTC reflecting LMN axonal hyperexcitability with reduction in SICI into a prognostic model showing ALS that patients manifesting both central and peripheral hyperexcitability have significantly reduced survival compared to ALS patients with one or no hyperactive parameters [71].

Additional work by Vucic and colleagues in symptomatic familial ALS (fALS) patients carrying the superoxide dismutase type 1 (SOD1) mutation revealed similar reduction in SICI and increased ICF in this specific cohort; asymptomatic SOD1 fALS patients also demonstrated significant reduction in SICI prior to development of clinical symptoms [73]. This finding suggests that cortical hyperexcitability may precede clinical symptoms of ALS, which are manifestations of predominantly LMN death, the putative “dying forward hypothesis” [73]. This technique has further evaluated the therapeutic effects of riluzole in significantly increasing SICI in patients with ALS treated with riluzole compared to untreated ones [74]. Ultimately, this foundational work suggests that (a) corticomotoneuronal hyperexcitability may precede the clinical syndrome of ALS and differentiate such patients from controls, (b) this hyperexcitability can be modulated by approved ALS therapies, and (c) use of such techniques as categorical and pharmacodynamic biomarkers in future clinical trials is warranted [64,75].

### 3.2. Peristimulus Time Histograms in ALS Patients

In addition to studying the MEP after applying TMS over a region of motor cortex, investigations led by Eisen and colleagues used the peristimulus time histogram (PSTH) technique to characterize the probability of firing a single voluntarily activated motor unit after a single motor cortex directed impulse. This approach allows for the investigation of a small pool of TMS-activated corticomotoneurons and their converging excitatory post-synaptic potentials on the spinal motor neuron, which leads to changes in the firing probability of the respective motor unit [76]. Normally, when a voluntarily recruited single motor unit encounters a TMS-induced corticomotoneuron volley, an increased probability of motor unit firing occurs with a latency of 20 ms after the stimulation; this is the primary peak, which reflects the compound excitatory post-synaptic potential (EPSP) [77].

In normal aging, there is a linear decline of the EPSP amplitude, while in purely LMN disorders there is no change to the EPSP [77]. In ALS, multiple PSTH patterns have been demonstrated, likely reflecting differing time-points across disease progression. While most ALS patients exhibit a reduced or absent EPSP, another subset demonstrates higher than expected EPSP amplitudes for age [77]. Later studies demonstrated a delay in the primary peak onset, increased temporal dispersion and more EPSP peak sub-components in the post-TMS corticomotoneuron volley period within ALS subjects [78,79]. These changes are theorized to reflect asynchronous temporal summation of the descending corticomotoneuron volley as well as increased repetitive firing of a hyperactive corticomotoneuronal pool in ALS [79]. This method provides an additional way to study progression of UMN degeneration in ALS, and possibly serve as a pharmacodynamic biomarker of therapeutic target engagement in clinical trials.

## 4. Extra-Motor Manifestations of MND

### 4.1. Autonomic Dysfunction

While motor system physiology has received much attention in ALS and other MNDs, increasing understanding of comorbid dysautonomia has helped reveal the multisystem involvement of these diseases. Patient-reported dysautonomia symptoms are frequently reported in ALS [80], with gastrointestinal, urinary, and sudomotor dysfunction described through the validated Parkinson’s Disease-Autonomic Dysfunction (SCOPA-AUT) scale at rates higher than healthy controls [81]. Objective testing also reveals abnormalities in ALS patients with both length-dependent and non-length-dependent decreases in quantitative sweat axon reflex testing (QSART) [80,82], decreased heart rate variability to deep breathing [80,82,83], decreased heart-rate Valsalva ratios [83], and increased resting heart rates [81]. Other groups have described decreased sympathetic skin responses in ALS compared with healthy controls [84,85], reflecting dysfunction of a somatosensory sympathetic reflex arc terminating with sweat gland depolarization generated in response to noxious stimulation or psychologic stress [86]. Post-mortem studies have also described progressive loss of neurons within the intermediolateral cell columns contained within the thoracic spinal cord known to participate in the sympathetic arc of the peripheral nervous system [87]. Given the descriptive efforts discussed here, a longitudinal natural history study of autonomic dysfunction in ALS patients is underway (Clinical Trial ID NCT05747937).

### 4.2. High-Density Electroencephalography and Cognitive Dysfunction

Considering the known association of ALS with frontotemporal dementia, as well as the growing understanding of cognitive manifestations of ALS, it is important to assess the function of distributed brain networks in this condition. Resting state high-density EEG (HDEEG) demonstrates increased connectivity across the frontocentral cerebrum of cognitively normal ALS patients thought to reflect pathologic hyperconnectivity within the Salience and Default Mode networks [88]. This abnormal hyperconnectivity may contribute to executive and attentional impairments seen in ALS, manifested by poorer performance on the Stroop Color–Word Interference neuropsychologic test and reduced auditory attentional discrimination of abnormal sonic tones by auditory mismatch negativity late cortical responses [89]. These attention network abnormalities seen in ALS have been further characterized by differences in the sustained attention to response task (SART) test, in which a subject must perform a motor task correctly when presented with the appropriate visual stimulus, often in the paradigm of being presented with various numbers on a computer screen and clicking the mouse only for specific digits when presented [90]. This Go-No-Go SART can be performed while undergoing EEG, and specific event-related potentials can differentiate ALS patients from healthy controls, even when their performance on the test itself is similar [90,91].

### 4.3. Magnetoencephalography and Cognitive Dysfunction

Magnetoencephalography (MEG) has also been used to explore MND electrophysiology, as high-definition resolution of cortical beta band synchronization has been found to have a role in movement preparation and execution, which is dysfunctional in ALS [92]. ALS patients demonstrate augmented beta band desynchronization in the bilateral motor cortex during movement preparation phase, followed by a delayed rebound of beta power after movement completion which is thought to reflect local cortical hyperexcitability [92]. Like studies with EEG, resting-state MEG in ALS patients reveals diffusely increased connectivity throughout cortical networks, particularly within posterior cingulate cortex and its connections to the motor cortex [93]. These tools can reliably differentiate ALS patients from healthy controls, sub-stratify disease subtypes based exclusively on unique cortical network abnormalities, and possibly serve as both categorical and pharmacodynamic biomarkers of ALS cortical dysfunction in clinical trials [94]. What remains to be seen is whether comorbid psychiatric disorders, including generalized anxiety, may cloud diagnostic utility with these tools, as such disorders also affect these widely distributed cognitive networks [95].

### 4.4. Somatosensory Dysfunction

ALS and other MNDs also demonstrate changes in the somatosensory system, ranging from patient-reported sensory complaints to neurophysiologic sensory abnormalities and evidence of sensory neural tissue pathology. Specifically in the brain at the cortical sensory neuron level, median nerve-stimulated sensory evoked potential (SEP) N20p-P25p peaks have been found elevated in ALS patients compared to healthy controls [96]. Interestingly, amplitudes greater than 8 µV of such median SEP peaks in ALS patients appear to be associated with shorter survival [96]. The normal late high-frequency SEP following the N20 is believed to represent the post-synaptic inhibitory response of GABAergic neurons in primary somatosensory cortex [97]. Compared to healthy controls, patients with ALS symptoms longer than 2 years demonstrate significant reduction of this late high-frequency burst, suggesting disinhibition of the somatosensory cortex as disease progresses [97].

In the PNS of patients with ALS, the sensory nerve axon has also been found to have abnormalities. Multiple groups have shown reduced sensory nerve action potential (SNAP) amplitudes and sensory nerve conduction velocities in both upper and lower extremities of patients, which worsen with disease progression [98,99,100]. In a cohort of ALS patients undergoing sural nerve biopsies, the numbers of large caliber myelinated fibers were reduced, and regenerating nerve fiber clusters were more frequent than in healthy control samples [100], with confirmation of these findings in later studies [101]. Small fiber abnormalities have also been described in patients with ALS. Although some groups have found reduced intra-epidermal nerve fiber densities on skin biopsy in ALS patients compared to age-matched controls, others have not [102,103,104]. Nonetheless, most epidermal nerve fiber studies report increased swellings and spheroid formations along small fibers suggestive of sensory axonal transport defects in ALS [102,103,104].

## 5. Discussion and Conclusions

ALS and related MNDs remain areas of intense research aimed at unraveling their complex pathophysiologies and developing effective therapies. The advancements in and application of electrophysiologic techniques, including MUNE, MUNIX, MScanFit, NPI, F-wave investigations, EIM, SFEMG and TMS, have significantly enhanced our understanding of both UMN and LMN dysfunction. These methods offer promise not only in improving diagnostic accuracy and stratification but also in serving as sensitive biomarkers of disease progression and therapeutic target engagement. Furthermore, evaluating the cognitive, autonomic, and sensory nervous systems reveals the multisystem nature of ALS.

For example, the clinician faced with a patient manifesting UMN-predominant ALS may use one of several electrodiagnostic techniques assessing LMN dysfunction to determine whether it is present subclinically and vice versa to secure a diagnosis of ALS. In addition, one could imagine the application of such electrophysiologic techniques in ALS clinical drug trials to better: (1) assist in stratifying subjects by specific UMN, LMN, and even non-motor electrical findings, (2) track objective neurophysiologic changes rather than patient-reported scales, and (3) assess target engagement or therapeutic effect rather than rely only on subject disability and/or mortality. We observed a recurring limitation of publications describing these various neurophysiologic techniques used in MND patients to usually differentiate them from healthy individuals rather than from disease mimics, which is by far the more common clinical scenario encountered by neurologists. Further research is needed to test the limits and help these tools to find their place in routine clinical practice.

Nonetheless, the integration of these advanced neurophysiologic tools into clinical trials has the potential to streamline the evaluation of therapeutic efficacy, ultimately accelerating the development of effective treatments for ALS and other MNDs. Continued research in this field is essential to refine these techniques and validate their clinical utility, bringing us closer to improving patient outcomes and quality of life.

## Figures and Tables

**Table 1:** Consensus criteria on LMN Needle EMG findings in ALS.

	El Escorial (1994) [7]	Awaji (2008) [9]	Gold Coast (2020) [10]
EMG Evidence of Chronic Denervation and Reinnervation	Definite *All of the following:* (1) Reduced motor unit recruitment (2) Large MUAP amplitudes and durations Probable *Any one or more of the following:* (1) Reduced motor unit recruitment with unstable MUAPs (2) Large MUAP amplitude and duration with unstable MUAP (3) Reduced MUNE Possible ^§^	*Any one or more of the following:* (1) Decreased motor unit recruitment (2) Increased MUAP amplitude and duration (3) Unstable and complex MUAP	*Any one or more of the following:* (1) Increased MUAP amplitude (2) Increased MUAP duration *Supportive but not obligatory findings:* (1) Polyphasic MUAPs (2) Unstable MUAPs
EMG Evidence of Active Denervation	Definite (1) Fibrillation potentials in muscle with chronic neurogenic injury meeting the definite criteria above Probable (2) Fibrillation potentials in muscle with chronic neurogenic injury meeting the possible criteria above	*Any one or more of the following:* (1) Fibrillation and/or positive sharp waves (2) Fasciculation potentials only if the test muscle demonstrates chronic neurogenic injury as above AND if the fasciculations are complex and/or unstable	*Any one or more of the following:* (1) Fibrillation and/or positive sharp waves (2) Fasciculation potentials

^§^ Note that in the original El Escorial Criteria there were also findings on electromyography suggestive of possible lower motor neuron degeneration including: (1) either reduced recruitment, large MUAP potentials, fibrillations, or unstable MUAP alone; (2) polyphasic MUAP or increased single fiber density alone; (3) low amplitude MUAP if disease duration longer than 5 years; (4) low amplitude CMAPs; (5) CMAP change between proximal and distal sites of stimulation uniform along length of nerve; (6, 7) summarized as no evidence of definitive demyelinating changes; (8) no more than 20% decrement on 2 Hz repetitive nerve stimulation; (9) no more than 10% decrement in sensory nerve action potential amplitude or sensory conduction velocity; (10) complex repetitive discharges; and (11) absence of fasciculations [7]. Abbreviations: CMAP, compound motor action potential; MUAP, motor unit action potential.

## Data Availability

No new data were created or analyzed in the preparation of this review. Data sharing is not applicable to this article.
